# Effect of the pre-taper level of fatigue on the taper-induced changes in performance in elite swimmers

**DOI:** 10.3389/fspor.2024.1353817

**Published:** 2024-02-21

**Authors:** Quentin Bretonneau, Antonio Morales-Artacho, Robin Pla, Laurent Bosquet

**Affiliations:** ^1^ Laboratoire MOVE (UR 20296), Faculté des Sciences du Sport, Université de Poitiers, Poitiers, France; ^2^ Laboratoire Sport, Expertise and Performance (EA 7370), Institut Français du Sport (INSEP), Paris, France; ^3^ Fédération Française de Natation (FFN), Service Optimisation de la Performance, Clichy, France

**Keywords:** elite athletes, sport performance, training load, overtraining, taper strategy, sleep, nocturnal core body temperature, swimming

## Abstract

**Introduction:**

In swimming, performance gains after tapering could be influenced by the pre-taper level of fatigue. Moreover, this level of fatigue could be associated with sleep. This study aimed to assess (1) the effect of tapering on performance according to the pre-taper level of fatigue in swimmers and (2) the association between sleep and pre-taper level of fatigue.

**Methods:**

Physiological, psychological and biomechanical profiles were evaluated in 26 elite swimmers on 2 occasions to estimate the pre-taper level of fatigue: at *T*0 and *T*1, scheduled respectively 10 and 3 weeks before the main competition. Sleep quantity and quality were also evaluated at *T*0 and *T*1. Race time was officially assessed at *T*0, *T*1 and during the main competition. The level of significance was set at *p *≤ .05.

**Results:**

Fourteen swimmers (17 ± 2 years) were allocated to acute fatigue group (AF) and 12 swimmers (18 ± 2 years) to functional overreaching group (F-OR). From *T*1 to the main competition, performance was improved in AF (+1.80 ± 1.36%), while it was impaired in F-OR (−0.49 ± 1.58%, *p* < 0.05 vs. AF). Before taper period, total sleep time was lower in F-OR, as compared to AF. Conversely, the fragmentation index was higher in F-OR (*p *= .06). From wakefulness to sleep, body core temperature decreased in AF but not in F-OR.

**Discussion:**

Performance gain after tapering was higher in AF swimmers than in overreached. Moreover, pre-taper sleep was poorer in overreached swimmers, which could contribute to their different response to the same training load. This poorer sleep could be linked to a lower regulation of internal temperature.

## Introduction

According to the mathematical model proposed by Banister and Fitz-Clarke (1), performance in sports such as swimming is largely determined by the difference between the level of physical fitness and the level of fatigue. Thus, reaching a peak performance for a given event, such as the Olympic Games, requires to identify the intervention modalities that reduce as much as possible the level of fatigue, without altering the level of physical fitness. The main strategy used by coaches, also known as taper, consists in decreasing the training load during the last 2–4 weeks before the competitive event. In their meta-analysis, Bosquet et al. (2) established that the taper strategy that was the most efficient in high-performance athletes was a gradual 40%–60% decrease in training volume over a two-week period, while maintaining exercise intensity and frequency. The weighted average performance gain was 1.9%, which is considerable in the context of high-performance sport, since the smallest enhancement of performance that has a substantial effect on the probability to win a medal has been estimated to about one-third of the typical variation of performance in competition, which is approximately 0.5–1% in swimming (3).

However, Bosquet et al. (2) also reported an important heterogeneity, performance gains being occasionally very different between athletes. According to the model proposed by Banister and Fitz-Clarke (1), the key factor to consider in individualizing this strategy is the pre-taper level of cumulated fatigue: the higher it is, the greater the reduction in training load should be. This hypothesis has been confirmed by the mathematical modeling study by Thomas and Busso (4), and by several experimental studies in endurance or team sports (5–7). However, data obtained in elite swimmers are scarce (8).

The mechanisms involved in the taper-induced improvement in performance capacity are numerous (2). However, the quality and quantity of sleep appear to play a pivotal role in the recovery process. In fact, beyond its ability to rest the body, sleep also stimulates anti-inflammatory, antioxidant and anabolic processes that will facilitate cellular and tissue repair, and contribute to an improved recovery (9). If our knowledge about sleep, recovery and performance is continuously increasing, data about its association with pre–taper fatigue and performance changes are still lacking.

The primary purpose of this study was to assess the efficiency of tapering on performance in elite swimmers according to the pre-taper level of fatigue. We hypothesized that taper-induced performance gains would be lower in swimmers with the highest level of fatigue. A secondary purpose was to investigate the association between the sleep and the pre-taper level of fatigue. We hypothesized that swimmers with the highest level of fatigue have a poorer quality and quantity of sleep.

## Materials and methods

### Protocol

**Figure 1:**
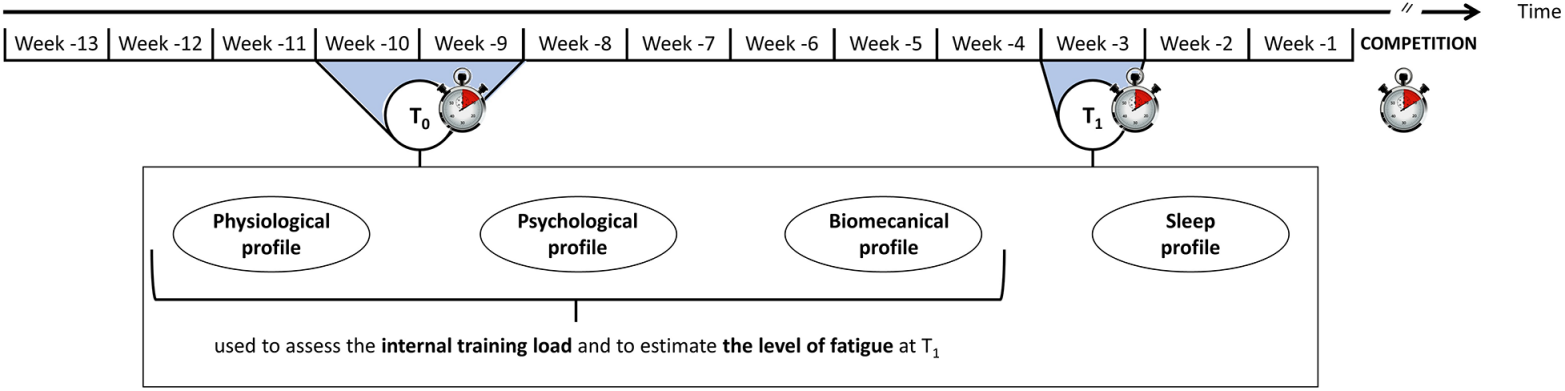
Experimental protocol.

The protocol started 13 weeks before the competition of interest and ended just after this competition (Figure 1). Physiological, psychological and biomechanical profiles were evaluated on two occasions to estimate the pre-taper level of fatigue: at *T*0 (a two-week baseline period scheduled ten and nine weeks before the competition of interest) and *T*1 (a one-week period scheduled three weeks before the competition). Sleep profile was also evaluated during these two periods. Considering changes in physiological, psychological and biomechanical profiles between *T*0 and *T*1, two groups were formed a posteriori: one group with an acute level of fatigue at *T*1 (i.e., before the taper period) and one group with a higher level of fatigue. Taper-induced changes in performance were compared between the two groups, as well as their pre-taper sleep quality and quantity. The study was conducted in three national training centres between January 2021 and December 2021. The competitions of interest were French National 1 championship, French Junior championship or French National 2 championship, according to the centre.

### Subjects

Assuming the use of an independent samples *t*-test (athletes in acute fatigue vs. athletes in functional overreaching), the power analysis indicated that eight athletes per group would be needed to have an 80% chance of obtaining a significant difference (*p* < 0.05) in taper-induced gain in performance (i.e., decrease in race time). According to this analysis based on the results of Aubry et al. (5), at least 16 athletes had to be recruited. Due to the uncertainty associated with the distribution of the participants between the groups (i.e., dependent on the level of fatigue), the risk of withdrawal from the study and the risk of a lack of competition or performance, it was agreed to double this number and recruit 30 participants with an equal distribution between male and female.

Fifteen male swimmers (age: 17.8 ± 2.2 years old; body height: 1.80 ± 0.07 m; body mass: 71.2 ± 7.5 kg; personal best performance: 88.4 ± 3.1% of the world record) and fifteen female swimmers (age: 16.4 ± 1.2 years old; body height: 1.70 ± 0.05 m; body mass: 59.5 ± 9.0 kg; personal best performance: 88.9 ± 2.5% of the world record) were included in the study. All of them were considered Tier 3 swimmers according to the classification by McKay et al. (10) (i.e., highly trained/national). Three male swimmers voluntarily withdrew from the study and one female swimmer was excluded due to injury and her inability to fully follow the protocol. The final sample size of this multicentric study was twelve male swimmers (age: 17.9 ± 2.3 years old; body height: 1.80 ± 0.08 m; body mass: 71.0 ± 8.4 kg; personal best performance: 88.0 ± 3.0% of the world record) and fourteen female swimmers (age: 16.4 ± 1.3 years old; body height: 1.69 ± 0.05 m; body mass: 59.6 ± 9.3 kg; personal best performance: 89.1 ± 2.5% of the world record).

The committee for the protection of the persons of Ile de France (i.e., the French ethical committee for biomedical research) has reviewed and approved the protocol of this study (N° ID-RCB: 2020-A03559-30 and N° CNIL/MR-001: 2220344). Moreover, the protocol was conducted according to recognized ethical standards and national/international laws. All the participants (or the legal representatives for the minor participants) gave their written informed consent to participate in the study.

### Procedure

#### Performance

Performance was defined by the race time measured during official competitions scheduled and certified by the French Swimming Federation. The race time was measured with an Omega system (Swiss Timing LTD, Corgémont, Suisse) and exported from the official website of the federation. Performances were evaluated at *T*0 and *T*1 (±7 days) and during the main competition. For each swimmer, we focused on the race (stroke and distance) for which the personal best time over the career was closest to the world record (in %, i.e., PB Career). We also focused on the race for which the personal best time over the protocol was closest to the world record (i.e., PB Protocol; Supplementary Material). The studied races were therefore specific to each athlete (Supplementary Material).

#### External training load

The external training load was assessed during the ten weeks leading up to the competition. After each swimming and dryland training session, the athletes had to indicate on a logbook the duration of the session (in min) and the rating of perceived exertion (RPE) determined from the CR-10 scale. The swimmers were familiarized with the scale at the beginning of the protocol. Furthermore, this scale was available in the logbook. The training load was obtained by multiplying the duration of the session and the RPE (11, 12) and was expressed in arbitrary unit. Load, duration and RPE data from swimming sessions (i.e., SS_load_, SS_duration_, SS_RPE_) et dryland sessions (i.e., DS_load_, DS_duration_, DS_RPE_) were analysed. Data from combined sessions (i.e., CS_load_, CS_duration_ and CS_RPE_), which represents the sum of swimming and dryland sessions, were also analysed. Throughout the protocol, the coaches were invited to maintain their usual strategy about the regulation of the external training load (e.g., number, duration and intensity of swimming and dryland training sessions during development and taper periods). Changes in load, duration and RPE during the taper period were calculated as follows (Eq. 1):

(1) $$\text{\%}\;\mathrm{decrease}=\frac{A-(B\times 14)}{B\times 14}\times 100$$

where *A* was the sum of each day of the 14-day taper and *B*, the mean of the daily three weeks preceding the taper.

The kinetics of the taper was also studied. Before the analysis, day-to-day load, duration and RPE data were smoothed with a running average including the d-day value and the values from the previous six days. The signal obtained was analysed using linear and exponential regression models and coefficients of determination were compared.

#### Internal training load

The physiological, psychological and biomechanical profiles were evaluated at *T*0 and *T*1 (Figure 1) to estimate the internal response of swimmers to training.

##### Physiological profile

The physiological profile was defined from heart rate (HR) measured at rest, during and after a standardized constant submaximal exercise. Heart rate was measured continuously at a frequency of 1 Hertz with a wrist-worn heart rate monitor using photoplethysmography (Polar Unite, Polar Electro Oy, Kempele, Finland). The monitor was worn on the non-dominant wrist and had to be particularly tight during exercise (13).

Resting heart rate was measured every morning during *T*0 and *T*1. Data were recorded for 5 min, in a dark space, before getting out of bed. The athletes had to be lying down and had to minimize movements before and during the measurement.

Exercise heart rate was measured every day during *T*0 and *T*1. The athletes had to swim for 6 min and cover the same distance each day. This distance was determined during the first days of *T*0. RPE had to be 3 on the CR10 scale (i.e., moderate perceived intensity) and HR had to be between 120 and 160 bpm. Once the distance was determined, it was imposed throughout the protocol.

At the end of the exercise, the swimmers had to remain vertical, leaning on a water lane and had to limit their movements for 3 min. Heart rate recovery after exercise was characterized by the difference between the HR measured at the end of the exercise and that measured 60 s after (i.e., Δ60).

These parameters were retained for analysis due to their sensibility to overtraining (14, 15) and their reproducibility (16–18).

##### Psychological profile

The psychological profile was defined from the profile of mood states questionnaire [POMS; (19)]. The resilience of the athlete was also assessed with the Connor-Davidson resilience scale [CD-RISC; (20)].

The POMS is a 65-item questionnaire that provides information on six specific mood states: vigor, depression, fatigue, anger, anxiety and confusion. The difference between the scores of vigor and fatigue was calculated to obtain the energy index (EI). Considering their sensibility to overload- and taper-induced changes in performance, vigor, fatigue and EI were retained for subsequent analysis (15). This questionnaire was administrated once at *T*0 and *T*1.

The CD-RISC is a 25-item questionnaire validated for the elite athlete population (21) and was administered once at the beginning of the protocol. It was used to determine the degree of confidence we can have in the result of the POMS questionnaire (22).

##### Biomechanical profile

The biomechanical profile was defined from the force measured during a 10-s front crawl tethered swimming test. The test was performed twice a day for three consecutive days at *T*0 and twice a day on a single day at *T*1. After a standardized 20-min warm-up, the swimmers performed two trials of 10-s front crawl tethered swimming, without breathing and at maximal intensity. The trials were separated by 5 min of passive recovery.

During the test, a 5-m stainless steel non-stretch cable was linked to the swimmer via a belt placed around his pelvis on one side, and to a mono-axial force transducer (Laumas®, IP68 acquisition frequency 256 Hz) on the other side. This transducer had a measurement range of 0–2,000 N, with a sensitivity of 2 mV/V, for a combined error of measurement of ±0.02%. The sensor was fixed to a hanger parallel to the surface of the water, and the cable rolled around a pulley to make sure that the force developed by the swimmer was applied along the load axis of the sensor. The hanger and the pulley were fixed on a stainless plate, which was fixed to the bar below the starting block with four clamps. The device was fixed to the same starting block to be sure that the angle between the water and the cable was the same for each trial. The sensor was connected to a Wi-Fi transmitter (SG-Link®200, IP68) and a computer placed near the swimming pool received data.

The signal obtained during the test represented the force produced by the swimmer as a function of time. This signal was manually segmented into cycles (one cycle represented the force produced by one action of each arm). As recommended, the first cycle was removed from analyses (23). From the remaining cycles, the mean force was calculated, as well as the mean impulsion (i.e., the integral of the force over the time interval). The data from the two daily trials were averaged.

#### Sleep profile

The sleep profile was defined from self-reported and objective sleep measurements.

The self-reported sleep measurements provided information on the chronotype, the sleep habits, the daytime sleepiness and the perceived sleep quality of athletes.

The chronotype of athletes was assessed once at the beginning of the protocol using the Horne and Ostberg morningness-eveningness questionnaire [19 questions, 16–86 score range; (24)]. A score between 16 and 30 indicated “definite evening., 31–41 “moderate evening”, 42–58 “intermediate type”, 59–69 “moderate morning” and 70–86 “definite morning”.

The sleeping habits of athletes were assessed once at *T*0 using the Pittsburgh sleep quality index [PSQI, 0–21 score range; (25)]. A score higher than five suggested a poor quality of sleep.

The daytime sleepiness was assessed once at *T*0 and *T*1 using the Epworth sleepiness scale [0–24 score range; (26)]. This questionnaire was composed of eight questions linked to the risk of dozing in different contexts. A score between 0 and 5 indicated lower normal daytime sleepiness, 6–10 normal daytime sleepiness, 11–12 mild excessive daytime symptoms, 13–15 moderate excessive daytime symptoms and 16–24 severe excessive daytime symptoms.

Perceived sleep quality was assessed every morning during *T*0 and *T*1 using the Spiegel questionnaire [six questions, 0–30 score range; (27)]. A mean score below 18 suggested sleep disturbances and a score below 15 represented a severe alert. A few minutes after getting up, the athletes had to answer the questionnaire integrated into their logbooks. They also had to specify information about the bedtime (i.e., the time they go to bed, not necessarily to sleep), the light out time (i.e., the time they turned off the light or their smartphone with the intention of sleeping) and the wake-up time.

Sleep quantity and quality were assessed every night during *T*0 and *T*1, with a connected headband and a triaxial wrist-worn accelerometer.

A few minutes before turning off the light to sleep, the swimmers had to equip themselves with a connected headband (Dreem 1, Dreem, New York, USA). This headband contained sensors that measured the different waves emitted by the brain during the night, which provided information on the architecture of sleep. Total sleep time (TST) was measured, as well as wake after sleep onset (WASO), rapid eye movement (REM) and non-rapid eye movement (NREM) duration. During the NREM period, light sleep (N1), deeper sleep (N2) and deepest NREM sleep (N3) duration were also measured. Latency of sleep onset (SOL), REM, N2 and N3 were assessed, as well as the number of awakenings and the sleep efficiency. The first two sleep cycles were also studied (start and end times, duration of N3 in each cycle).

In addition to the headband, the athletes had to wear an accelerometer at the non-dominant wrist to quantify the nocturnal movements (ActiGraph wGT3X-BT, ActiGraph LLC, Pensacola, USA). Counts were measured on the three axes. Furthermore, movement index and fragmentation index were assessed.

Core body temperature was measured at *T*0 and *T*1 during at least one night in the middle of the week. The athletes had to ingest an encapsulated thermometer (length: 17.7 mm, diameter: 8.9 mm) around 6 p.m. (e-Celsius Performance, BodyCap, Caen, France). The temperature was measured every 2 min and data were collected the following morning via a monitor using telemetry (e-Viewer Performance, BodyCap, Caen, France). The temperature could be measured for two successive nights, depending on the transit of the athlete. A first analysis focused on the data measured between 9 p.m. and 7 a.m. A second analysis focused on the data measured between 1 h before the light out to sleep and 7 h after. The minimum temperature and the time at which it was measured were also studied.

### Categorization of fatigue

Depending on the variations of the profiles and the underlying variables between *T*0 and *T*1, the athletes were considered either in acute fatigue (AF group) or in functional overreaching (F-OR group). The distribution was based on the strategy previously developed by Vachon et al. (7) (i.e., the number and the magnitude of negative changes). More specifically, athletes were included in the AF group when multiple small to moderate negative changes were highlighted and no more than one large negative change was observed, a negative EI being considered as such (i.e., scenario 1). Athletes were included in the F-OR group when at least one large negative change was observed on two distinct profiles or more (i.e., scenario 2).

For variables measured several times at *T*0 and *T*1 (e.g., heart rate), the magnitude of the difference was determined from Cohen's *d*, calculated as follows (Eq. 2):

(2) $$d=({\bar{X}}_{T0}-{\bar{X}}_{T1})/pooled\;standard\;deviation\;(SD)$$

with,

$$\mathrm{pooled}\;\mathrm{SD}=\surd (\left(\left(SD_{T0}^{2}\times (n_{T0}\text{–}1)+{\mathrm{SD}}_{T1}^{2}\times (n_{T1}\text{–}1))/(n_{T0}+n_{T1}\text{–}2\right)\right))$$

and *n* the number of measures

For variables measured several times at *T*0 and a single time at *T*1 (e.g., the force developed during the tethered swim test), the magnitude of the difference was determined from the *Z* score, calculated as follows (Eq. 3):

(3) $$Z\;=({\bar{X}}_{T1}-{\bar{X}}_{T0})/SD_{T0}$$

For variables measured a single time at *T*0 and at *T*1 (e.g., vigor), the magnitude of the difference was expressed according to the smallest worthwhile change (SWC), calculated as follows (28) (Eq. 4):

(4) $$\mathrm{SWC}=0.2\times \mathrm{between}\mathrm{athlete}\;SD_{T0}$$

The thresholds used to determine the magnitude of the difference are detailed for each method in Table 1.

**Table 1:** Thresholds used to determine the magnitude of the difference depending on the statistical procedures.

	Method based on *d* Cohen	Method based on *Z* score	Method based on SWC
Positive change or trivial negative change	│*d* Cohen│ < 0.2	│*Z* score│ <0 .67	│Δ│ < 1 SWC
Small negative change	≥0.2 and <0.5	≥0.67 and <0.96	≥1 SWC and <3 SWC
Moderate negative change	≥0.5 and <0.8	≥0.96 and <1.34	≥3 SWC and <6 SWC
Large negative change	≥0.8 and <1.2	≥1.34 and <2.33	≥6 SWC and <10 SWC
Very large negative change	≥1.2	≥2.33	≥10 SWC

SWC, smallest worthwhile change.

### Statistical analysis

Standard statistical methods were used to calculate the means and SD. Normal Gaussian distribution was verified by a Shapiro-Wilk test. An independent samples *t*-test, or when appropriate a Wilcoxon test, was used to test the null hypothesis (1) that AF and F-OR groups were not different at *T*0, (2) that external training loads during taper were not different between groups and (3) that changes in performance between *T*1 and the competition of interest were not different between groups. A two-way factorial analysis of variance (group × period) with repeated measures on the period factor was performed to test the null hypothesis that measures were not different between groups and periods. A specific three-way analysis of variance (group × period × time) was performed for the core body temperature and the counts measured by accelerometry. Compound symmetry, or sphericity, was checked by the Mauchly test. When the assumption of sphericity was not met, the significance of *F* ratios was adjusted according to the Greenhouse–Geisser procedure when the epsilon correction factor was <0.75, or according to the Huynh–Feldt procedure when the epsilon correction factor was >0.75 to control for a type I error. Multiple comparisons were made with the Bonferroni *post hoc* test. The magnitude of the effect was assessed by the Hedges' *g* (g). The magnitude of the effect was considered small (0.2 < *g* < 0.5), moderate (0.5 < *g* < 0.8), or large (*g* > 0.8). Pearson's product-moment correlation, or when appropriate Spearman rank-order correlation, was used to test the null hypothesis of an absence of association between variables and/or their variations. We considered a correlation over 0.90 as very high, between 0.70 and 0.89 as high and between 0.50 and 0.69 as moderate (29). The significance level was set at *p* < .05 for all analyses. All the calculations were made with Statistica (StatSoft, Tulsa, USA) and Excel (Microsoft, Redmond, USA).

## Results

### AF and F-OR groups

Individual variations of the physiological, psychological and biomechanical parameters between *T*0 and *T*1 are presented in Table 2. Fourteen swimmers were allocated to the AF group (age: 16.8 ± 1.7 years old; body height: 1.75 ± 0.10 m; body mass: 63.4 ± 10.9 kg; personal best performance: 87.9 ± 2.7% of the world record), while the 12 other swimmers were allocated to the F-OR group (age: 17.5 ± 2.1 years old; body height: 1.74 ± 0.06 m; body mass: 66.5 ± 10.10 kg; personal best performance: 89.5 ± 2.6% of the world record).

**Table 2:** Individual variations (Δ**)** of physiological, psychological and biomechanical parameters between *T*0 and *T*1.

	Physiological profile			Psychological profile				Biomecanical profile		Decision making	
Participant	Δ resting heart rate	Δ exercise heart rate	Δ delta 60	Δ fatigue	Δ vigor	Δ EI	EI *T*1 < 0	Δ force	Δ impulsion	Scenario	Group
1	—	—	—	—	↓	—	—	—	—	1	AF
2	—	—	↑↑↑↑	↑↑	↓↓↓↓	↓↓↓	✓	—	—	2	F-OR
3	—	—	—	—	—	—	—	—	↓	1	AF
4	↑↑	—	—	—	—	—	—	—	—	1	AF
5	↑	—	—	—	—	—	—	↓↓↓	—	1	AF
6	—	↓↓↓↓	—	—	—	—	—	—	—	1	AF
7	—	—	↑	—	—	—	—	↓↓↓	—	1	AF
8	↑↑	↓↓	—	—	—	—	—	—	—	1	AF
9	—	↓	↑↑↑	—	—	—	—	—	—	1	AF
10	—	↓↓↓↓	—	↑↑	—	↓	✓	↓	—	2	F-OR
11	—	—	—	—	↓↓↓↓	↓↓↓	—	↓↓↓	—	2	F-OR
12	—	↓↓↓↓	↑↑↑↑	—	—	—	✓	↓↓↓↓	—	2	F-OR
13	—	↓↓↓↓	—	↑↑	↓↓↓↓	↓↓↓↓	✓	↓↓↓↓	—	2	F-OR
14	—	↓↓↓	—	—	—	—	—	↓↓↓	↓↓↓↓	2	F-OR
15	—	↓↓↓↓	—	—	—	—	—	↓	↓	1	AF
16	—	—	↑↑↑↑	—	—	—	—	↓↓	↓↓↓	2	F-OR
17	↑↑↑	↓	—	—	↓↓↓↓	↓↓↓	—	↓↓↓	↓↓↓	2	F-OR
18	↑↑	↓↓↓↓	—	—	↓	—	—	↓↓↓	—	2	F-OR
19	—	↓↓↓	↑↑	—	↓↓↓↓	↓↓	✓	—	—	2	F-OR
20	↑	—	—	—	—	—	—	—	↓↓	1	AF
21	—	—	↑↑↑↑	—	↓↓↓	↓	—	—	—	2	F-OR
22	↑	—	—	—	↓↓	—	✓	—	—	1	AF
23	↑↑	↓	—	—	—	—	✓	—	—	1	AF
24	—	↓↓↓↓	↑↑	—	—	—	—	—	—	1	AF
25	↑↑↑	—	↑↑↑↑	—	—	—	✓	—	—	2	F-OR
26	—	↓↓↓↓	—	—	↓↓	—	—	—	—	1	AF

AF, acute fatigue; F-OR, functional overreaching; EI, energy index; —, positive change or trivial negative change; one arrow, small negative change; two arrows, moderate negative change; three arrows, large negative change and four arrows, very large negative change (large and very large negative changes are highlighted by grey-shaded cells).

### Training load and taper characteristics

**Figure 2:**
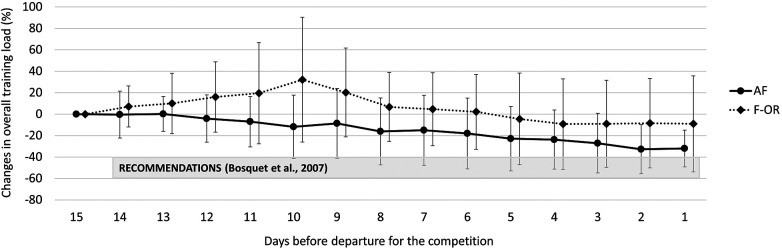
Changes in overall training load during the taper period in acute fatigue (AF) and functional overreaching (F-OR) groups.

Overall training load and its decomposition between dryland- and swimming-training load did not change from *T*0 to *T*1 and were similar between groups. As illustrated in Figure 2, we observed a 30 ± 20% linear decrease of overall training load 12 days before the competition in AF (*r*^2^ = 0.97), while there was a 23 ± 32% exponential decrease 10 days before the competition in F-OR (*r*^2^ = 0.98). We found no difference between groups, no matter it was training duration (−26 ± 15% vs. −18 ± 21% in AF and F-OR groups, respectively), or training RPE (−9 ± 26% vs. −12 ± 28% in AF and F-OR groups, respectively). If we focus on training load decomposition, we found no difference between groups regarding swimming-training load (−28 ± 27% in average). In contrast, dryland-training load decreased by 30 ± 38% in AF, and increased by 38 ± 71% in F-OR.

### Performance

A total of 213 official swimming performances were obtained during the study, corresponding to an average of 8 performances per participant. Changes in race time from *T*0 to *T*1 were not different between groups (−0.36 ± 1.79% vs. +0.25 ± 2.28% for the race associated with PB Career and −0.85 ± 1.25% vs. +0.19 ± 2.17% for the race associated with PB Protocol in AF and F-OR groups, respectively). In contrast, changes in race time from *T*1 to the main competition differed between groups, since performance for the race associated with PB Career improved by 1.80 ± 1.36% in AF, while it decreased by 0.49 ± 1.58% in F-OR (*p* < 0.05 vs AF). We found no difference in performance changes for the race associated with PB Protocol (−2.10 ± 1.09% vs. −0.49 ± 1.86% in AF and F-OR groups, respectively).

### Sleep architecture and core body temperature

Sleep characteristics at *T*0 and *T*1 are detailed in Table 3 for both groups. Sleep latency, wake after sleep onset and wake-up time were not different between these two periods and between groups. Swimmers from F-OR exhibited a lower total sleep time, a tendency toward a higher fragmentation index and a higher day-time sleepiness, whatever the period. Bedtime and light out time increased from *T*0 to *T*1, without difference between groups.

**Table 3:** Sleep characteristics at *T*0 and *T*1 in AF and F-OR groups. Data are reported as mean ± SD.

	*T*0		*T*1	
	AF group	F-OR group	AF group	F-OR group
Questionnaires				
Bed time (hh:min)	21:37 ± 00:41	21:53 ± 00:47	21:59 ± 01:06^a^	22:09 ± 00:48^a^
Light out time (hh:min)	22:10 ± 00:37	22:13 ± 00:38	22:28 ± 00:56^a^	22:35 ± 00:34^a^
Wake-up time (hh:min)	06:57 ± 00:23	06:43 ± 00:28	06:58 ± 00:35	06:36 ± 01:05
Time from light out to wake-up (min)	526 ± 41	516 ± 34	506 ± 48^a^	486 ± 56^a^
Score at the Spiegel questionnaire	21.3 ± 3.4	20.0 ± 3.0	21.4 ± 3.0	20.7 ± 3.3
Score at the Epworth sleepiness scale	8.4 ± 4.6	10.3 ± 2.6^b^	7.9 ± 2.5	10.9 ± 3.3^b^
Connected headband				
Sleep onset latency (min)	20 ± 12	21 ± 6	22 ± 10	18 ± 15
Sleep onset time (hh:min)	22:28 ± 00:38	22:27 ± 00:39	22:52 ± 00:58^a^	22:57 ± 00:59^a^
Wake after sleep onset (%)	25.5 ± 10.2	20.4 ± 9.1	22.6 ± 8.8	20.3 ± 12.2
Total sleep time (TST, min)	08:15 ± 00:39	07:47 ± 00:37^b^	07:55 ± 00:48^a^	07:08 ± 00:56^a^^,^^b^
Sleep efficiency (%)	91.2 ± 3.0	91.6 ± 2.1	91.5 ± 2.4	91.5 ± 4.5
Number of awakenings/hour from light out to wake-up	3.4 ± 0.7	2.9 ± 1.3^b^	3.7 ± 0.5	2.9 ± 1.0^b^
REM (min)	116.5 ± 22.8	112.2 ± 14.4	121.3 ± 20.0	104.3 ± 20.4
REM (% TST)	23.7 ± 4.4	23.9 ± 2.4	25.5 ± 2.9	23.1 ± 4.1
REM latency (min)	96.6 ± 28.4	82.7 ± 16.5	88.4 ± 29.0	77.2 +±17.7
N1 duration (min)	30.7 ± 8.0	23.4 ± 6.6^b^	30.1 ± 5.4	22.5 ± 8.4^b^
N1 (% TST)	6.2 ± 1.0	5.0 ± 1.6^b^	6.3 ± 0.9	5.2 ± 1.6^b^
N2 duration (min)	207.5 ± 31.4	187.9 ± 33.8^b^	198.8 ± 28.6	167.4 ± 32.9^b^
N2 (% TST)	42.7 ± 4.8	40.3 ± 6.4	41.8 ± 4.7	39.3 ± 7.8
N2 latency (min)	4.6 ± 2.0	5.0 ± 4.3	5.0 ± 1.1	4.3 ± 2.2
N3 duration (min)	125.6 ± 17.8	135.3 ± 24.4	122.6 ± 22.4	124.3 ± 33.7
N3 (% TST)	25.4 ± 4.1	30.0 ± 6.0	25.7 ± 4.3	29.4 ± 7.2
N3 latency (min)	14.6 ± 6.4	13.7 ± 3.6	15.1 ± 3.6	17.1 ± 13.5
NREM (min)	376.4 ± 36.3	355.0 ± 31.5	355.1 ± 35.3^a^	323.5 ± 40.9^a^
NREM (% TST)	76.3 ± 4.4	76.1 ± 2.4	74.5 ± 2.9	76.9 ± 4.1
Start time of the first cycle (hh:min)	22:26 ± 00:35	22:31 ± 00:48	22:52 ± 01:01^a^	22:45 ± 00:41^a^
End time of the first cycle (hh:min)	23:46 ± 00:30	23:51 ± 00:51	00:12 ± 01:01^a^	00:10 ± 00:35^a^
Duration of N3 in the first cycle (min)	48.9 ± 6.8	50.7 ± 5.5	45.7 ± 8.8	53.4 ± 12.0
Start time of the second cycle (hh:min)	23:41 ± 00:35	23:47 ± 00:38	00:07 ± 01:11^a^	00:19 ± 00:29^a^
End time of the second cycle (hh:min)	01:13 ± 00:34	01:26 ± 00:49	01:48 ± 01:02^a^	02:02 ± 00:33^a^
Duration of N3 in the second cycle (min)	46.7 ± 12.8	52.4 ± 8.4	44.7 ± 11.4	50.7 ± 8.2
Accelerometer				
Total counts 3 axes/min from light out to wake-up	318 ± 171	304 ± 96	330 ± 134	271 ± 71
Movement index (%)	16.3 ± 4.2	17.6 ± 5.1	16.9 ± 4.1	16.5 ± 3.9
Fragmentation index (%)	11.5 ± 3.2	14.0 ± 4.7	12.1 ± 4.4	15.2 ± 3.0

^a^ Significant period effect.

^b^ Significant group effect.

**Figure 3:**
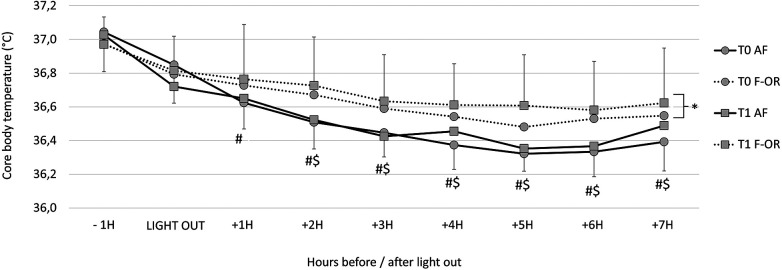
Core body temperature kinetics during sleep in acute fatigue (AF) and functional overreaching (F-OR) groups. For the corresponding group: #, different from −1h; $, different from Light out and *, different from *T*0.

The kinetics of core body temperature during sleep are detailed in Figure 3. The average temperature was not different between *T*0 and *T*1 in AF (36.48 ± 0.23°C vs. 36.50 ± 0.23°C, respectively), while it increased in F-OR (from 36.61 ± 0.24°C to 36.67 ± 0.28°C). It is worth noting that during the hour preceding bedtime, temperature decreased by 0.23 ± 0.26°C in AF and by 0.14 ± 0.22°C in F-OR (*p* = .07 for the group effect). The nadir of temperature kinetics was lower in AF than in F-OR (36.12 ± 0.15°C vs. 36.31 ± 0.25°C, respectively). The time at which the lowest temperature was measured was not different between periods and groups (02h53 ± 02h07), as was the duration after the light out (+04h30 ± 01h44).

### Association between relevant variables

**Figure 4:**
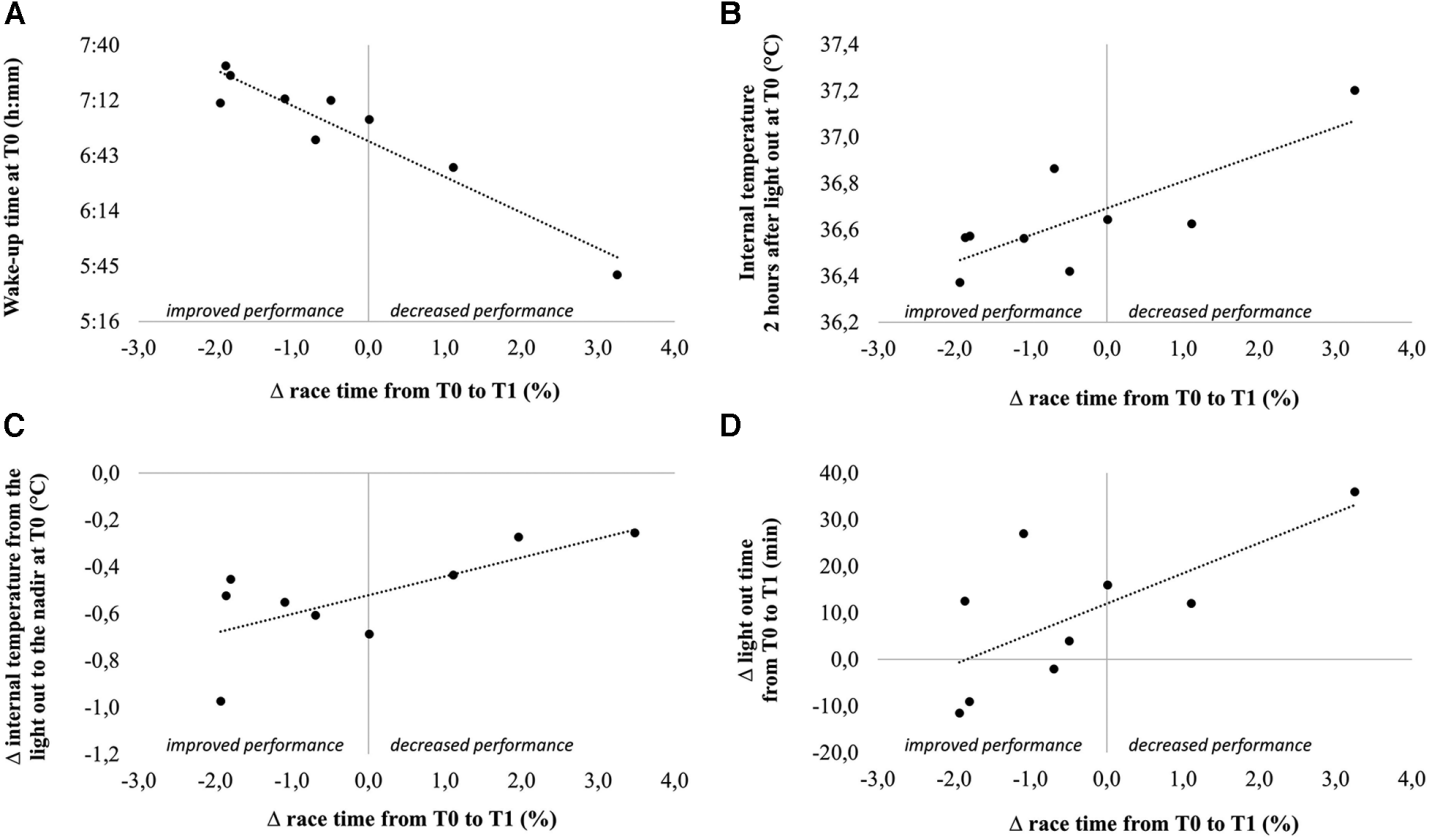
Correlations between change in performance from *T*0 to *T*1 and wake-up time at *T*0 (**A**), internal temperature 2 h after light out at *T*0 (**B**), change in internal temperature from the light out to the nadir at *T*0 (**C**) and change in light out time from *T*0 to *T*1.

Change in race time from *T*0 to *T*1 was associated with multiple variables assessed at *T*0, such as the wake-up time (*r* = −0.95), temperature measured two and three hours after the light out (*r* = 0.78 and *r* = 0.68, respectively) and the difference in temperature between the light out and the nadir (*r* = 0.70). Change in race time from *T*0 to *T*1 was also associated with the change in light out time (*r* = 0.69) (Figure 4).

Interestingly, at *T*0 the number of awakenings per hour of sleep was associated with the temperature measured at the light out (*r* = 0.69), and with the change in temperature during the hour preceding the light out (*r* = 0.68). The change in number of awakenings per hour of sleep from *T*0 to *T*1 was associated with the change in CS_load_ (*r* = 0.75) and SS_load_ (*r* = 0.68). The change in N3 latency between the two periods was associated with the change in N3 duration (*r* = −0.81).

## Discussion

The primary purpose of this study was to assess the efficiency of tapering on performance in elite swimmers according to the pre-taper level of fatigue. Since our population was made up of Tier 3 swimmers (i.e., highly trained/national level) and the final competition of the protocol was the main objective of the competitive season, the approach we adopted consisted of describing what was done in the different training groups rather than imposing a specific taper strategy. Nevertheless, the recommendations of the meta-analysis by Bosquet et al. (2) were presented to all coaches at the start of the protocol. Two groups corresponding to two different levels of fatigue were formed a posteriori from the objective and subjective responses of swimmers to the training period: the AF group (*n* = 14) and the F-OR group (*n* = 12). Their respective pre-taper training load was not different, thus suggesting that any difference between groups originated from a different response to the same training load, and not from an overload period before the taper in F-OR. In contrast, we observed a different taper strategy between groups, particularly in terms of duration. The consequence was an overall reduction in training load that was lower than the recommendations (30 ± 20% during 12 days in AF and 23 ± 32% during 10 days in F-OR, while the recommendation is 40%–60% during 14 days). It was also surprising to note that the decrease in training load of F-OR swimmers was lower than AF swimmers, while they had a higher level of fatigue and a shorter taper period. According to the mathematical simulations of Thomas and Busso (4), the opposite would have been expected. This discrepancy highlights the complexity of taper implementation in elite athletes, where the ability of the coach to accurately estimate the level of fatigue, his confidence in the taper process, the preferences of swimmers and other contextual aspects can interfere and make it difficult to fulfill recommendations. Whatever the cause of this difference in taper strategy between groups, it is evident from our results that they impacted performance variation. In fact, the taper strategy of AF, which was closest to the recommendations and probably more adapted to the level of fatigue of the group, led to an average improvement of 1.82%, which is consistent with expected gains (2, 4). Conversely, we observed a decrease by 0.49% in F-OR, which is both in agreement with the literature (5–7), and expected if we consider the discrepancy between the level of fatigue to be recovered and the decrease in training load used during the taper period. This highlights the importance of identifying strategies other than the manipulation of the training load to decrease the level of fatigue (e.g proactive recovery methods) and verifying their possible additive effect in the most fatigued swimmers. Vachon et al. (30) published data supporting this idea in young elite rugby players, but that needs to be tested in swimmers.

A secondary purpose of this study was to assess the association between the sleep and the pre-taper level of fatigue. Our main observation was that sleep quality and quantity of the F-OR swimmers were poorer before the taper, as compared to AF swimmers. Briefly, F-OR swimmers showed a shorter TST, a tendency toward a higher fragmentation index during the night, and a higher daytime sleepiness. Moreover, core body temperature decreased between waking and sleep phases in AF swimmers but not in F-OR swimmers. In F-OR swimmers furthermore, average core body temperature during sleep was higher at *T*1 than at *T*0. However, these differences should be interpreted with caution as the temperature was measured only for a single night (or two consecutive nights in 18% of cases).

Otherwise, change in performance from *T*0 to *T*1 was associated with wake-up time at *T*0 (the earlier the wake up, the lower the performance gain) and with the change in light out time from *T*0 to *T*1 (the swimmers who improved the most were those who shifted the most their bedtime to the early evening). Temperature kinetics at the start of the night, which is known to play a major role in sleep onset, was also associated with the number of awakenings per hour of sleep.

Altogether, these observations underscore the importance of identifying strategies that may improve sleep quality and quantity, especially before the taper period. Beyond the decrease in training load, sleep education is a strategy that should be tested in swimmers, especially to make them more aware of the importance of a regular and early bedtime. Strategies that may impact the kinetics of body temperature at the start of the night, such as thermoregulating mattresses (31, 32), cryostimulation (33) or cold-water immersion (34, 35) should also be considered. It is now important to test their possible additive effect with the taper, particularly in swimmers whose level of fatigue is such that taper alone will not be enough to fully recover and obtain a peak performance. It should be kept in mind however that these strategies need to be anticipated, particularly if we consider the difficulty of elite athletes to adopt new lifestyle habits (36), and also the difficulty of coaches to modify strategies that have been designed gradually and have demonstrated their effectiveness.

## Pratical Recommendations

The purpose of this study was to assess the efficiency of tapering on performance in elite swimmers according to the pre-taper level of fatigue. Our results showed that taper-induce changes in performance were lower in overreached swimmers than in swimmers with acute level of fatigue. Furthermore, our results showed that sleep quality and quantity were poorer in swimmers with the highest level of fatigue. Therefore, it could be relevant for coaches and their technical staff (1) to assess and improve if necessary the quality and quantity of sleep before an overload period to prevent a high level of accumulated fatigue and (2) to follow the recommendations on the reduction of the training load during the taper period to observe benefits on performance.

## Data Availability

The data is available from the corresponding author through direct contact via their email address.

## D-DAY consortium members

- Laboratoire MOVE (UR 20296), UFR STAPS Poitiers, Université de Poitiers, France: Arc-Chagnaud Coralie, Bosquet Laurent, Bretonneau Quentin, Delpech Nathalie, Dugué Benoit, Dupuy Olivier, Enéa Carina, Pichon Aurélien, Tanneau Maxence et Theurot Dimitri.
- Institut Pprime, UPR 3346, CNRS – Université de Poitiers – ISAE-ENSMA, France: Couvertier Marien, Decatoire Arnaud, Monnet Tony et Samson Mathias.
- Institut de recherche biomédicale des armées (IRBA), Brétigny-sur-Orge, France: Sauvet Fabien.
- Unité Vigilance, Fatigue, Sommeil et Santé Publique (VIFASOM, EA7330), Université de Paris, Hôtel Dieu, Paris, France: Sauvet Fabien.
- Laboratoire SEP (EA 7370), INSEP, France: Morales-Artacho Antonio, Nédelec Mathieu, Pasquier Florane, Poignard Mathilde et Ruffault Alexis.
- Fédération Française de Natation, Clichy, France: Pla Robin.
- Laboratoire Culture, Sport, Santé, Société (C3S, EA4660), UFR STAPS Besançon, Université de Franche-Comté, France: Bouzigon Romain.
- Société Aurore Concept, Noisiel, France: Bouzigon Romain.
- Société Inside the Athletes 3.0, Besançon, France: Bouzigon Romain.
