# Supplementary material for: Effect of the pre-taper level of fatigue on the taper-induced changes in performance in elite swimmers
Source: Front Sports Act Living. 2024 Feb 21;6:1353817. doi: 10.3389/fspor.2024.1353817 (PMC10915210; doi:10.3389/fspor.2024.1353817)
Supplement: Supplementary file 1 [file Table1.docx]

**Supplementary Table 1: Race for which the personal best time over the career and over the protocol was closest to the world record.**

| Participant | Race for which  the personal best time over the **career** was closest to the world record (WR) | Personal best time  (in % WR) | Race for which  the personal best time over the **protocol** was closest to the world record | Personal best time  (in % WR) |
| --- | --- | --- | --- | --- |
| 1 | 400m freestyle | 91.41 | 400m freestyle | 91.41 |
| 2 | 100m breaststroke | 91.73 | 100m breaststroke | 91.73 |
| 3 | 400m freestyle | 90.27 | 400m freestyle | 90.27 |
| 4 | 100m butterfly | 89.56 | 100m butterfly | 89.56 |
| 5 | 200m breaststroke | 85.29 | 100m breaststroke | 84.49 |
| 6 | 200m breaststroke | 90.18 | 200m breaststroke | 90.18 |
| 7 | 800m freestyle | 85.63 | 800m freestyle | 85.63 |
| 8 | 100m freestyle | 89.73 | 100m freestyle | 89.73 |
| 9 | 200m breaststroke | 85.22 | 200m breaststroke | 85.22 |
| 10 | 50m breaststroke | 88.71 | 100m freestyle | 88.54 |
| 11 | 100m freestyle | 86.07 | 200m freestyle | 85.31 |
| 12 | 800m freestyle | 91.98 | 800m freestyle | 91.98 |
| 13 | 400m freestyle | 89.79 | 1500m freestyle | 89.01 |
| 14 | 400m individual medley | 92.48 | 400m individual medley | 91.95 |
| 15 | 200m breaststroke | 92.23 | 200m breaststroke | 92.04 |
| 16 | 100m backstroke | 92.35 | 200m freestyle | 90.44 |
| 17 | 800m freestyle | 92.19 | 800m freestyle | 92.19 |
| 18 | 100m freestyle | 88.83 | 100m freestyle | 87.34 |
| 19 | 400m freestyle | 86.04 | 400m freestyle | 86.04 |
| 20 | 50m backstroke | 85.41 | 50m backstroke | 84.82 |
| 21 | 400m individual medley | 86.11 | 100m butterfly | 83.49 |
| 22 | 400m individual medley | 85.42 | 400m individual medley | 84.78 |
| 23 | 200m individual medley | 84.04 | 200m individual medley | 83.24 |
| 24 | 200m freestyle | 87.82 | 200m freestyle | 87.82 |
| 25 | 400m freestyle | 87.22 | 400m freestyle | 87.22 |
| 26 | 50m butterfly | 87.82 | 50m butterfly | 87.82 |
